# Supplementary material for: Matching sensor ontologies through siamese neural networks without using reference alignment
Source: PeerJ Comput Sci. 2021 Jun 18;7:e602. doi: 10.7717/peerj-cs.602 (PMC8237319; doi:10.7717/peerj-cs.602)
Supplement: Supplemental Information 1 [file peerj-cs-07-602-s001.zip › 259/onto.html]

Author: Nick Knouf <nknouf@mit.edu>  
Contributor: Antoine Zimmermann <antoine.zimmermann@inrialpes.fr>, Jérôme Euzenat,   
Date: 08/06/2005  
Version: $Id: onto-exp.rdf,v 1.10 2008/05/27 14:40:02 euzenat Exp $

## Classes

**http://www.w3.org/1999/02/22-rdf-syntax-ns#List** (, *)*


**http://xmlns.com/foaf/0.1/Person** (, *)*


**http://xmlns.com/foaf/0.1/Organization** (, *)*


**uqwz** (, *)*
:   - #sbqgzga [0 1]

    **sqdsq** (, *)*
    :   - #sqndsqgy [0 1]
        - #dznbaln [0 1]
        - #sbqgzga [0 1]

        **PBdgcs** (, *)*
        :   **zajdhqs** (, *)*
            :   - #dznbaln [1 1]
                - #zsbdgz [0 1]
                - #sddnsnqdb [0 1]
                - #dszbnz [0 1]
                - #sqndsqgy [1 1] *#dzshyuua*
                - #zand [1 1]
                - #sdsqddz [0 1]

                **dzajndsq** (, *)*
                :   - #iNi4b8XV *#dzqndbzq*

                **sqxsqkd** (, *)*
                :   - #iNi4b8XV *#dzqndbzq*
                    - #TfniY *#dcsqdcsqd*

                **zdqssqdb** (, *)*
                :   - #PrSGUs *#deqdxcsqcsq*
                    - #zadzjadns [0 1] *#cexQ*
                    - #dzajj [0 1]
                    - #sqdndvb [0 1]

            **qsdsnbsqd** (, *)*
            :   - #dznbaln [1 1]

                **zdzaudbd** (, *)*


                **scds** (, *)*


                **dcsqdsq** (, *)*
                :   - #sqdndvb [0 1]
                    - #sdsqddz [0 1]
                    - #dznbaln [1 1]

                    **ZikPzsz** (, *)*


                    **ZGKW1E** (, *)*

                **zeadza** (, *)*
                :   - #zand [1 1]
                    - #dznbaln [1 1]
                    - #zdsnsqdv [1 +oo]

            **sqdsopq** (, *)*
            :   - #dzayue [0 1]
                - #dznbaln [1 1]

                **RJbGVM** (, *)*
                :   - #zand [1 1]
                    - #dzayue [1 1]
                    - #sxqsnbvsq [1 1]
                    - #sqndsqgy [1 1] *#dzshyuua*
                    - #ndsbzh [0 1]
                    - #zsbdgz [0 1]

                    **hazdn** (, *)*


                    **Be** (, *)*


                    **fGfFm** (, *)*


                    **wFuU** (, *)*

                **dzqndbzq** (, *)*
                :   - #dsazdjz [0 1] [0 1]

                **vccfsq** (, *)*
                :   - #zand [1 1]
                    - #dzayue [1 +oo]
                    - #sznsbq [1 1]

                **dcsqdcsqd** (, *)*
                :   - #zand [1 1]
                    - #sqnbyz [1 1]

                **deqdxcsqcsq** (, *)*
                :   - #zand [1 1]
                    - #zassdzadb [1 1]

            **zdazsx** (, *)*
            :   - #zand [1 1]
                - #dznbaln [1 1]
                - #dzbn [1 1]
                - #sqndsqgy [1 1]

                **xsqlknk** (, *)*


                **zqdzqzai** (, *)*

            **wxsdxqsdh** (, *)*


            **dqzdxdcsqj** (, *)*
            :   - #zand [1 1]
                - #dznbaln [1 1]
                - #hsgiuyza [1 1]
                - #sqndsqgy [1 1] *#dzshyuua*
                - #ndsbzh [0 1]

                **EwQu** (, *)*
                :   **xhNJuGU** (, *)*


                    **UWmlX** (, *)*


                    **FPVijs** (, *)*
                    :   **cnNCBO** (, *)*
                        :   **ZVxsNo** (, *)*


                            **jZVxsNo** (, *)*

                        **Lluuk** (, *)*
                        :   **sdcsqhyz** (, *)*


                            **QMgwFB** (, *)*


                            **YeLgZJu** (, *)*


                            **LXtNU** (, *)*

                    **XaWa** (, *)*
                    :   **FkmaDDV** (, *)*

                **azzauhuie** (, *)*
                :   - #zandsbh [0 1]

                    **pjDVyIr** (, *)*

        **dscdscg** (, *)*

**BeXCIG** (, *)*
:   - #dszabdza [1 1] *http://www.w3.org/2001/XMLSchema#string*
    - #dsza *http://www.w3.org/2001/XMLSchema#string*
    - #sdxsndxsqg *http://www.w3.org/2001/XMLSchema#string*
    - #sddnsnqdb [0 1]
    - #dszbnz [0 1]
    - #sqdnbs [0 1]
    - #contributions *#RJbGVM*

    **qsdsquj** (, *)*


    **wxVRZcIXbbz** (, *)*

**cexQ** (, *)*
:   - #dszabdza [1 1]
    - #sdnsbh *#izxnquo*
    - #dsza [0 1]
    - #sqnzkzn [0 1]
    - #zdjznzn [0 1]

    **zqedzbx** (, *)*


    **RcrfZK** (, *)*


    **qHdl** (, *)*


    **izVyrw** (, *)*

**qzddj** (, *)*
:   - #zadszabnds [0 1] *http://www.w3.org/2001/XMLSchema#string*
    - #zdnzadh [0 1] *http://www.w3.org/2001/XMLSchema#string*
    - #zdzndh [0 1] *http://www.w3.org/2001/XMLSchema#string*

**izxnquo** (, *)*
:   super: *http://xmlns.com/foaf/0.1/Organization*  

    - #dszabdza [1 1]
    - #dsza [1 1]
    - #qzd [0 1]

    **YpNk** (, *)*


    **zauio** (, *)*


    **BriFsw** (, *)*
    :   **MyRFOvC** (, *)*


        **zadazxn** (, *)*


        **SNNu** (, *)*


        **SLoVq** (, *)*

**dsqdbz** (, *)*
:   super: *http://www.w3.org/1999/02/22-rdf-syntax-ns#List*  

    - http://www.w3.org/1999/02/22-rdf-syntax-ns#first [1 1] *http://xmlns.com/foaf/0.1/Person*
    - http://www.w3.org/1999/02/22-rdf-syntax-ns#rest [1 1] (*#dsqdbz* | )

**dnqsb** (, *)*
:   - #sqnhdbdj [1 1]
    - #mkalnshsq [1 1]

**dzshyuua** (, *)*
:   - #zdsnzsdh [1 1] *http://www.w3.org/2001/XMLSchema#gYear*
    - #asndzsd [0 1] *http://www.w3.org/2001/XMLSchema#gMonth*
    - #dznadzh [0 1] *http://www.w3.org/2001/XMLSchema#gDay*

## Properties

**http://www.w3.org/1999/02/22-rdf-syntax-ns#first**: http://www.w3.org/1999/02/22-rdf-syntax-ns#List -> \_ *()*


**http://www.w3.org/1999/02/22-rdf-syntax-ns#rest**: http://www.w3.org/1999/02/22-rdf-syntax-ns#List -> http://www.w3.org/1999/02/22-rdf-syntax-ns#List *()*


**iNi4b8XV**: #PBdgcs -> #dzqndbzq *()*


**TfniY**: #sqdsq -> #sqdsopq *()*


**PrSGUs**: #zdqssqdb -> #deqdxcsqcsq *()*


**contributions**: #BeXCIG -> #RJbGVM *()*
:   **YuEma**: #qsdsquj -> #hazdn *()*

**qzd**: http://www.w3.org/2002/07/owl#Thing -> #qzddj *()*


**zadzjadns**: #zdqssqdb -> #cexQ *()*


**sdnsbh**: #cexQ -> http://xmlns.com/foaf/0.1/Organization *()*


**zandsbh**: #sqdsq -> http://www.w3.org/2002/07/owl#Thing *()*


**sbqgzga**: #uqwz -> #dsqdbz *()*
:   **zand**: \_ -> \_ *()*


    **dzajj**: \_ -> \_ *()*


    **zdnzqnd**: #dscdscg -> \_ *()*

**hsgiuyza**: #dqzdxdcsqj -> #izxnquo *()*


**EzGNplyLBiS**: #sqdsopq -> \_ *()*
:   **sxqsnbvsq**: #RJbGVM -> #qsdsquj *()*


    **sznsbq**: #vccfsq -> #dzajndsq *()*


    **sqnbyz**: #dcsqdcsqd -> #sqxsqkd *()*


    **zassdzadb**: #deqdxcsqcsq -> #zdqssqdb *()*

**sqndsqgy**: (*#sqdsq* | *#zqedzbx*) -> #dzshyuua *()*


**sqdndvb**: (*#zdqssqdb* | *#dcsqdsq*) -> http://xmlns.com/foaf/0.1/Organization *()*


**sddnsnqdb**: (*#sqdsq* | *#BeXCIG*) -> #zauio *()*


**dzbn**: (*#zdazsx* | *#scds*) -> #BriFsw *()*


**zdjznzn**: (*#sqdsq* | *#zqedzbx*) -> #qzddj *()*


**dzayue**: #sqdsopq -> #dnqsb *()*

**http://purl.org/dc/elements/1.1/creator**\_ -> \_ *()*


**http://purl.org/dc/elements/1.1/contributor**\_ -> \_ *()*


**http://purl.org/dc/elements/1.1/description**\_ -> \_ *()*


**http://purl.org/dc/elements/1.1/date**\_ -> \_ *()*


**http://xmlns.com/foaf/0.1/firstName**\_ -> \_ *()*


**#lastName**\_ -> \_ *()*


**http://xmlns.com/foaf/0.1/name**\_ -> \_ *()*


**zdzbh** #sqdsq -> http://www.w3.org/2001/XMLSchema#string *()*


**dsqndbsqx** #sqdsq -> http://www.w3.org/2001/XMLSchema#string *()*


**dsqnhza** #sqdsq -> http://www.w3.org/2001/XMLSchema#string *()*


**sdxsndxsqg** #BeXCIG -> http://www.w3.org/2001/XMLSchema#string *()*


**sqdnbs** #dzqndbzq -> http://www.w3.org/2001/XMLSchema#string *()*


**sdsqddz**(*#zajdhqs* | *#dcsqdsq*) -> http://www.w3.org/2001/XMLSchema#string *()*


**sdqz**(*#wxsdxqsdh* | *#zdzaudbd*) -> http://www.w3.org/2001/XMLSchema#string *()*


**zdsnsqdv** #sqdsq -> http://www.w3.org/2001/XMLSchema#string *()*


**dszbnz** #sqdsq -> http://www.w3.org/2001/XMLSchema#string *()*


**dznbaln** #sqdsq -> http://www.w3.org/2001/XMLSchema#string *()*


**sqbdzg**(*#dzqndbzq* | *#sdcsqhyz* | *#zdazsx*) -> http://www.w3.org/2001/XMLSchema#string *()*


**zadzqbsdg** #sqdsq -> http://www.w3.org/2001/XMLSchema#string *()*


**dsqndsz** #sqdsq -> http://www.w3.org/2001/XMLSchema#string *()*


**zdnzdzh** #sqdsq -> http://www.w3.org/2001/XMLSchema#string *()*


**zdzdzd** #sqdsq -> http://www.w3.org/2001/XMLSchema#string *()*


**szdnzak** #zajdhqs -> http://www.w3.org/2001/XMLSchema#string *()*


**zadsznad** #BeXCIG -> http://www.w3.org/2001/XMLSchema#string *()*


**zqdszh** #sqdsq -> http://www.w3.org/2001/XMLSchema#string *()*


**sqkxndc** #sqdsq -> http://www.w3.org/2001/XMLSchema#language *()*


**dzandzah** #sqdsq -> http://www.w3.org/2001/XMLSchema#string *()*


**dszbgz** #sqdsq -> http://www.w3.org/2001/XMLSchema#string *()*


**dsq** #sqdsq -> http://www.w3.org/2001/XMLSchema#string *()*


**dzsndzah** #sqdsq -> http://www.w3.org/2001/XMLSchema#string *()*


**zdsznbadzagh** #sqdsq -> http://www.w3.org/2001/XMLSchema#string *()*


**dszabdza**\_ -> http://www.w3.org/2001/XMLSchema#string *()*


**dsza**\_ -> http://www.w3.org/2001/XMLSchema#string *()*


**dsazdjz** #sqdsopq -> http://www.w3.org/2001/XMLSchema#string *()*


**dzezd**(*#sqdsq* | *#zqedzbx*) -> \_ *()*
:   **ndsbzh** #sqdsq -> http://www.w3.org/2001/XMLSchema#string *()*


    **sqnzkzn**(*#sqdsq* | *#cexQ*) -> http://www.w3.org/2001/XMLSchema#string *()*


    **zsbdgz** #sqdsq -> http://www.w3.org/2001/XMLSchema#nonNegativeInteger *()*

**zdsnzsdh** #dzshyuua -> http://www.w3.org/2001/XMLSchema#gYear *()*


**asndzsd** #dzshyuua -> http://www.w3.org/2001/XMLSchema#gMonth *()*


**dznadzh** #dzshyuua -> http://www.w3.org/2001/XMLSchema#gDay *()*


**zdzndh** #qzddj -> http://www.w3.org/2001/XMLSchema#string *()*


**zdnzadh** #qzddj -> http://www.w3.org/2001/XMLSchema#string *()*


**zadszabnds** #qzddj -> http://www.w3.org/2001/XMLSchema#string *()*


**sqnhdbdj** #dnqsb -> http://www.w3.org/2001/XMLSchema#nonNegativeInteger *()*


**mkalnshsq** #dnqsb -> http://www.w3.org/2001/XMLSchema#nonNegativeInteger *()*

## Individuals

<rdf:List@ttp://www.w3.org/1999/02/22-rdf-syntax-ns#nil>

---

Generated by OWL2HTML
